# *Plasmodium reichenowi* EBA-140 merozoite ligand binds to glycophorin D on chimpanzee red blood cells, shedding new light on origins of *Plasmodium falciparum*

**DOI:** 10.1186/s13071-017-2507-8

**Published:** 2017-11-07

**Authors:** Agata Zerka, Radoslaw Kaczmarek, Marcin Czerwinski, Ewa Jaskiewicz

**Affiliations:** 1 Laboratory of Glycobiology, Hirszfeld Institute of Immunology and Experimental Therapy, Polish Academy of Sciences, Rudolfa Weigla 12, 53-114 Wroclaw, Poland; 2 Faculty of Physiotherapy and Physical Education, Opole University of Technology, 45-758 Opole, Poland; 3 Faculty of Biological Sciences, University of Zielona Góra, Szafrana 1, 65-516 Zielona Góra, Poland

**Keywords:** *Plasmodium reichenowi*, EBA-140 ligand, Glycophorin D, Host specificity, *Plasmodium* evolution

## Abstract

**Background:**

All symptoms of malaria are caused by the intraerythrocytic proliferation of *Plasmodium* merozoites. Merozoites invade erythrocytes using multiple binding ligands that recognise specific surface receptors. It has been suggested that adaptation of *Plasmodium* parasites to infect specific hosts is driven by changes in genes encoding *Plasmodium* erythrocyte-binding ligands (EBL) and reticulocyte-binding ligands (RBL). Homologs of both EBL and RBL, including the EBA-140 merozoite ligand, have been identified in *P. falciparum* and *P. reichenowi*, which infect humans and chimpanzees, respectively*.* The *P. falciparum* EBA-140 was shown to bind human glycophorin C, a minor erythrocyte sialoglycoprotein. Until now, the erythrocyte receptor for the *P. reichenowi* EBA-140 remained unknown.

**Methods:**

The baculovirus expression vector system was used to obtain the recombinant EBA-140 Region II, and flow cytometry and immunoblotting methods were applied to characterise its specificity.

**Results:**

We showed that the chimpanzee glycophorin D is the receptor for the *P. reichenowi* EBA-140 ligand on chimpanzee red blood cells.

**Conclusions:**

We propose that the development of glycophorin C specificity is spurred by the *P. falciparum* lineage. We speculate that the *P. falciparum* EBA-140 evolved to hijack GPC on human erythrocytes during divergence from its ape ancestor.

## Background

Malaria causes over a half million deaths per year, chiefly among children and pregnant women in sub-Saharan Africa, and most of the cases are caused by *Plasmodium falciparum* [1]. A closely related species, *Plasmodium reichenowi*, infects chimpanzees [2, 3]. Until 2009, only one *P. reichenowi* isolate was genetically characterised [4]. Due to the morphological similarity of these two species, it was initially suggested that *P. falciparum* originated from *P. reichenowi*, most likely by a single transfer from chimpanzees (*Pan troglodytes*) [5, 6] or evolved in bonobos (*Pan panicus*) [7]. However, it was previously shown that while humans cannot be infected by *P. reichenowi*, *P. falciparum* can infect chimpanzees, albeit without severe symptoms as seen in humans [3, 8].

Recently, the use of molecular tools for species identification to explore the diversity of *Plasmodium* species, have revealed new phylogentic species in great apes [6, 7, 9, 10]. As a result, in the old subgenus *Laverania*, six *Plasmodium* species were confirmed, of which *P. reichenowi*, *P. gaboni* and *P. billcollinsi* only infect chimpanzees, whereas *P. praefalciparum*, *P. adleri* and *P. blacklocki* only infect gorillas. Indeed, it was proposed that great apes are natural hosts to diverse *Plasmodium* species, including *P. falciparum* previously considered as strictly human-specific [6].

Sequencing of *Plasmodium* DNA from a large collection of ape fecal samples revealed the closest relative, and likely origin of human *P. falciparum*, is a clade of parasites found in the western gorillas [11]. This finding suggested a possible gorilla origin of human *P. falciparum*, in opposition to previous theories proposing chimpanzee-human transmission of *P. reichenowi-*related parasite. In particular, all known strains of *P. falciparum* circulating in humans nowadays resulted from a single cross-transmission event from gorilla to human [11]. These data suggested that ape-to-human transmission was possible, but an alternative theory that each parasite species evolved independently along with its host cannot be ruled out [12–15].

The genomic basis of the *P. falciparum* adaptation to human hosts was explored by sequencing the genomes of two closely related species, *P. reichenowi* and *P. gaboni*, parasitic in chimpanzees. While it was shown that the genomes of *P. falciparum* and *P. reichenowi* are remarkably similar, striking differences were found in the genes involved in red cell invasion, which determine host specificity [9, 16, 17]. Invasion of erythrocytes by *Plasmodium* parasites involves multiple ligands; merozoites bind to erythrocytes using proteins that belong to two families including erythrocyte-binding ligands (EBL) and reticulocyte-binding ligands (RBL) [18, 19]. It was suggested that changes in the sequence and arrangement of genes in the EBL and RBL family may be directly associated with *Plasmodium* adaptation to its host.

Several proteins in the *P*. *falciparum* EBL family recognise different human red blood cell receptors and thus enable the merozoite to gain entry through alternative invasion pathways. Four functional *P. falciparum* EBL proteins have been identified so far: erythrocyte-binding antigen-175 (EBA-175), erythrocyte-binding antigen-181 (EBA-181), erythrocyte-binding ligand-1 (EBL-1) and erythrocyte-binding antigen-140 (EBA-140) [20]. These proteins contain several conserved regions, such as Region II, which is involved in binding receptors on erythrocytes.

Recent results from the Malaria Genomic Epidemiology Network Project [21] demonstrated that resistance to malaria may be linked to the cluster of genes encoding human glycophorins, which are surface sialoglycoproteins of erythrocytes [22]. Thus, it may be argued that changes in the region of genes encoding glycophorins A, B, and possibly E, all of which may act as receptors for merozoite EBL proteins, arose as the result of strong evolutionary pressure exerted by *P. falciparum* on the human genome. These data emphasized the crucial role that EBL proteins and glycophorins play when merozoites burgle erythrocytes.

*Plasmodium falciparum* merozoite ligands that bind glycophorins show distinct binding behaviors, which result in different invasion pathways. The well-studied *P. falciparum* EBA-175 recognizes glycophorin A (GPA) [23–25], while EBA-140 [26–28] was shown to bind glycophorin C (GPC) [29–33], a minor erythrocyte sialoglycoprotein [32, 34]. Both EBA-140 and EBA-175 bind to erythrocytes in a sialic acid-dependent manner [35], but binding of EBA-140, also, requires that GPC is N-glycosylated [36]. EBA-175 recognises sialic acids present on clusters of O-linked glycans of glycophorin A (GPA). Homologs of merozoite EBL and RBL, including the EBA-140 protein, were identified in *P. reichenowi* [37, 38]. The amino acid sequences of the *P. falciparum* and *P. reichenowi* EBA-140 proteins are 81% identical, and the highest degree of similarity is seen within the binding region (Region II). However, the binding specificity of the *P. reichenowi* EBA-140 is still poorly understood. Both ligands require sialic acid for binding. Using the surface expression of human and chimpanzee EBA ligands on COS 7 cells, it was shown that the *P. falciparum* EBA-140 requires Neu5Ac, while its *P. reichenowi* counterpart requires Neu5Gc [8]. Thus, the difference in the binding specificity of these proteins may have arisen in response to the change of host “sialome” during the evolution of the human-specific *Plasmodium* species [39]. The human lineage lost the ability to turn Neu5Ac into Neu5Gc as a result of a mutation in the CMAH gene, which encodes the CMP-Neu5Ac hydroxylase. Thus, it has been hypothesized that the difference in sialic acid structure between humans and apes is the primary factor determining species-specific binding of malaria parasites [8, 39]. However, it was found that the EBA-175 ortholog from the chimpanzee-restricted parasites binds to human GPA with a similar affinity to that of *P. falciparum,* which suggests that the EBA 175-GPA interaction is probably not the sole determinant of *Plasmodium* host specificity [40]. Moreover, it was proposed that the interaction of the *P. falciparum* Rh5 RBL ligand with basigin on erythrocytes is a major determinant of host species tropism.

There is a general agreement that *P. falciparum* recognises GPC on human erythrocytes [29–33]. GPC is encoded in humans by the *GYPC* gene, which is unique among the glycophorin genes because it contains two separate translation initiation sites [41, 42]. This leads to the synthesis of (predominantly) GPC and its truncated form, called glycophorin D (GPD), which lacks the first 21 aa residues of GPC. It was shown that GPC, but not GPD, plays a role in erythrocyte invasion mediated by *P. falciparum* EBA-140 [29–32]. Also, GPC (but not GPD) contains N-glycan at the Asn8 residue, the presence of which seems to be necessary for receptor recognition [35, 36, 43]. The sequences of the *GYPC* gene homologs in six Hominidae species (human, chimpanzee, bonobo, gorilla, orangutan and white-cheeked gibbon) reveal a C to A transversion, which results in the emergence of a new start codon present only in humans [41]. Thus, humans are the only species that produce both GPC and GPD, with GPC being the major gene product. Therefore, it was suggested that GPD might be an ancestral receptor in nonhuman primates for *P. reichenowi-*like parasites, while GPC emerged as a new receptor in humans, targeted by *P. falciparum* [41].

To explain the role of GPC and GPD in *P. reichenowi* binding, we used the recombinant binding region (Region II) of *P. reichenowi* EBA-140 obtained in baculovirus expression vector system [44]. We demonstrate that the receptor for the *P. reichenowi* EBA-140 is probably the homolog of human glycophorin D on chimpanzee erythrocytes.

## Methods

### Erythrocytes

Chimpanzee (*Pan troglodytes)* blood was freshly collected on EDTA during non-experimental clinical veterinary practice in the Warsaw Zoological Garden. The blood was drawn from immobilised chimpanzee female during a medical intervention. This sample was used to perform diagnostic tests, and the intact remainder (0.5 ml) was used in our experiments.

### Flow cytometry analysis

The recombinant *P. reichenowi* EBA-140 Region II was incubated in phosphate buffered saline (PBS), pH 7.4 for 2 h at 4 °C with 3 × 10^5^ native and trypsin- and chymotrypsin- (Sigma-Aldrich, St. Louis, MO, USA) treated chimpanzee erythrocytes. The heat-denatured Region II was used as the negative binding control. The cells were washed three times with PBS and incubated for 1 h at 4 °C with rabbit serum (diluted 1:200) raised against the whole *P. falciparum* EBA-140 Region II [45]. The cells were washed three times with PBS and incubated for 45 min at 4 °C with FITC-conjugated swine anti-rabbit Ig antibody (DakoCytomation, Glostrup, Denmark) and analyzed for fluorescence intensity using flow cytometry (FACSCalibur, BD Biosciences, San Jose, USA). Mouse monoclonal antibody (MoAb) (clone 2G11 [46], diluted 1:500) recognizing human GPC and the chimpanzee GPD homolog was used as the binding control.

### Western blotting (overlay assay)

Proteins of native and enzyme-treated chimpanzee erythrocytes or human erythrocyte membranes were fractionated by SDS-PAGE using a 10% polyacrylamide gel according to the Laemmli method [47] and then transferred to a nitrocellulose membrane (Schleicher & Schuel, Dassel, Germany) according to the method of Towbin et al. [48]. The membranes were overlaid with the solution of *P. reichenowi* or *P. falciparum* recombinant Region II (100 μg/ml) in TBS overnight at room temperature. The bound Region II was detected with a mouse MoAb directed against the c-myc epitope (clone 9E10, ATCC, diluted 1:10). Erythrocyte GPD was detected on the blots with MoAb 2G11 [46] (diluted 1:500) recognizing N-terminal epitope (amino acid residues 14–20/14–18) and MoAb 1F6 [49] (diluted 1:50) recognizing C-terminal fragment (amino acids 110–115/89–94) on human GPC and GPD, respectively. The PageRuler Prestained Protein Ladder (Fermentas,Villnius, Lithuania) was used as a protein standard.

## Results

### Binding of the *P. reichenowi* EBA-140 region II to chimpanzee erythrocytes

**Fig. 1:**
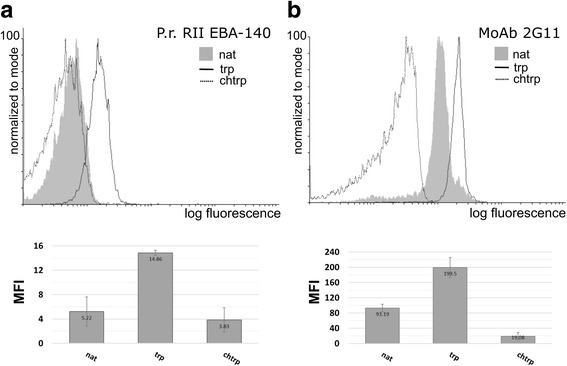
Flow cytometry analysis of the *P. reichenowi* EBA-140 Region II (RII) binding to native chimpanzee erythrocytes (nat) and erythrocytes treated with trypsin (trp) and chymotrypsin (chtrp) (**a**); the binding pattern of MoAb 2G11 recognizing GPC/GPD (**b**). *Abbreviation*: MFI, mean fluorescence intensity

Treatment of erythrocytes with proteolytic enzymes may influence binding of antibodies or other ligands in two ways: they can either degrade the receptor, thwarting the binding, or trim off only the proteins that shield the receptor, thus exposing its binding sites. We found that treatment of chimpanzee erythrocytes with chymotrypsin causes a slight decrease of the EBA-140 Region II binding (Fig. 1a). In contrast, the binding after trypsin treatment was markedly increased. We suggest that trypsin digestion removes proteins that sterically hinder the ligand-receptor interaction. MoAb 2G11, which specifically binds to human GPC/GPD showed a similar binding profile to chimpanzee erythrocytes (Fig. 1b), although the drop in binding after chymotrypsin treatment was more evident.

### Binding of EBA-140 region II to glycophorin D

**Fig. 2:**
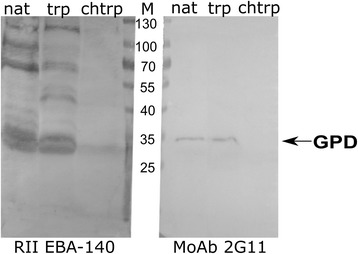
Western blotting analysis of the *P. reichenowi* EBA-140 Region II binding to chimpanzee erythrocyte proteins. Native (nat) and trypsin (trp) and chymotrypsin (chtrp)-treated chimpanzee erythrocytes; the recombinant Region II was detected with anti-myc MoAb 9E10; position of GPD was identified with MoAb 2G11. M, molecular weight marker

The binding of the *P. reichenowi* EBA-140 ligand Region II to chimpanzee erythrocyte proteins was evaluated by Western blotting (Fig. 2). The protein recognized by the Region II showed the apparent molecular weight of 35 kDa, which is a similar value to GPD recognized by MoAb 2G11. The binding of the EBA-140 Region II to the erythrocyte receptor was decreased after chymotrypsin treatment, but augmented by trypsin. These data suggest that the chimpanzee erythrocyte receptor for the *P. reichenowi* EBA-140 is the GPD homolog. The bands of higher molecular weight are GPC/GPD aggregates with other erythrocyte glycophorins, mostly GPA [22].

**Fig. 3:**
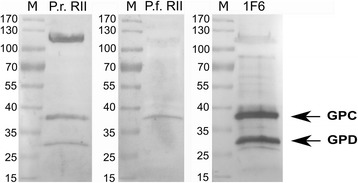
Western blotting analysis of the *P. reichenowi* and *P. falciparum* EBA-140 Region II binding to human erythrocyte membrane proteins. The recombinant Region II was detected with anti-myc MoAb 9E10; positions of GPD and GPC were identified with MoAb 1F6 [50]. M, molecular weight marker

Western blotting corroborated the specificity of the *P. reichenowi* EBA-140 Region II binding to GPD with human erythrocyte membranes (Fig. 3). We found that the *P. reichenowi* Region II binds to human GPC and its truncated form, GPD, while the homologous *P. falciparum* EBA-140 Region II binds only to GPC, as it was previously shown [32].

## Discussion

While genomes of *P. falciparum* and *P. reichenowi* are remarkably similar, large differences in the genes involved in red cell invasion (which determine host specificity), stand out against that conserved background [16]. It was shown that these genomes are essentially co-linear in the core central regions with a small number of significant differences. The most striking of these involve genes associated with red cell invasion. Within the five-member EBL family, EBA-165 is a pseudogene in *P. falciparum* but not in *P. reichenowi*, while EBL-1 has a substantial deletion in *P. reichenowi*. Similarly, of six RBLs in the *P. falciparum* genome, only three (Rh2b, Rh4, Rh5) have orthologues in *P. reichenowi*. The Rh2 locus seems to be, the most different between these two species. Notable differences can also be seen between the *rif* and *stevor* multigene families where the numbers are much lower in the human parasite [16].

Moreover, genes encoding other erythrocytic malaria antigens: MSP2 [50] and *var2CSA* (which is associated with malaria in pregnancy [51]), have shown recently extended polymorphism in *P. falciparum* that likely originated after the *P. reichenowi-P. falciparum* split [9]. Thus, it was hypothesized that changes in the sequence and arrangement of genes encoding erythrocytic stage antigens, especially the EBL and RBL proteins, may be directly associated with *Plasmodium* human adaptation. Additional support for this hypothesis comes from the evaluation of dimorphism of *P. falciparum* EBA-175 alleles [17] and human specificity of Rh5 ligand in receptor-binding preferences [40]. Explaining how this host-switch occurred in the evolution of *P. falciparum* lineage remains still one of the greatest challenges.

There is general agreement that GPC is the sialylated receptor on human erythrocytes for the *P. falciparum* EBA-140 [29–32]. Since EBA-140 ligand does not recognize GPA, which is also a highly glycosylated protein, it may be presumed that the GPC protein backbone is involved in the binding. Location of the binding site of EBA-140 close to N- and O-glycans at the N-terminal portion of GPC and lack thereof in its truncated form, GPD, may explain why the *P. falciparum* EBA-140 Region II does not bind to GPD.

**Fig. 4:**
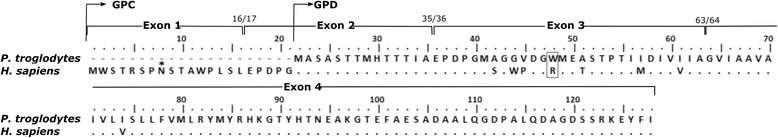
Amino acid sequence of human (*Homo sapiens*) GPC/GPD and chimpanzee (*Pan troglodytes*) GPD homolog (ENSPTRT 00000044133). Dots indicate identical amino acids; asterisk indicates N-glycan on GPC Asn8 residue

Until now, the erythrocyte receptor for the *P. reichenowi* EBA-140 remained unknown. To identify it, we used a soluble, recombinant Region II obtained in insect cells. We showed previously that the *P. reichenowi* Region II of EBA-140 binds specifically to chimpanzee erythrocytes in a sialic acid-dependent manner [44]. We found that binding of the EBA-140 Region II to GPD is markedly decreased by treatment of erythrocytes with chymotrypsin. Conversely, trypsin treatment enhances the binding, probably by removing proteins that sterically hinder access to GPD, while leaving GPD intact. This is in stark contrast to the effects of trypsin on human erythrocytes, which fail to bind the *P. falciparum* EBA-140 after digestion [32]. In our opinion, this discrepancy results from the presence of arginine (R27) in the human GPD (R48 in GPC), which introduces a trypsin digestion site, while the chimpanzee GPD contains tryptophan (W) at this position. The difference makes the chimpanzee GPD resistant to trypsin, but susceptible to chymotrypsin (Fig. 4). However, since tryptophan in chimpanzee GPD is followed by methionine, which is not a preferred residue at P1’ position, GPD may undergo only a partial digestion by chymotrypsin. As a result, residual binding of the *P. reichenowi* EBA-140 RII to erythrocytes after chymotrypsin treatment is detected by both flow cytometry and Western blotting experiments.

It was shown before that GPC, but not GPD plays a role in human erythrocyte invasion mediated by *P. falciparum* EBA-140 ligand [29–32]. Also, only GPC is N-glycosylated at the Asn8 residue, and the N-glycan seems to be necessary for receptor recognition [35, 36, 43]. It is presumed that *P. falciparum* EBA-140 ligand does not bind to human GPD [31], due to the lack of N-glycan. Thus, it may be speculated that the *P. falciparum* EBA-140 ligand lost the ability of its ancestor to bind GPD, but developed the specificity for GPC, involving its N-glycan and O-glycans.

## Conclusions

Our results show that the chimpanzee GPD is the receptor for the *P. reichenowi* EBA-140 on chimpanzee erythrocytes. Also, these results hint that development of EBA-140 GPC specificity may have helped *P. falciparum* to thrive in human erythrocytes when the human and chimpanzee lineages diverged from their ancestor.

## References

[1] WHO World Malaria 2014. WHO. 2015 [cited 2015 Oct 1]. Available from: http://www.who.int/malaria/publications/world_malaria_report_2014/en/. Accessed 9 Mar 2017.

[2] Escalante AA, Ayala FJ. Phylogeny of the malarial genus *Plasmodium*, derived from rRNA gene sequences. Proc Natl Acad Sci USA. 1994;91:11373–7. 10.1073/pnas.91.24.11373 PMC45233 7972067

[3] De Nys HM, Calvignac-Spencer S, Boesch C, Dorny P, Wittig RM, Mundry R (2014). Malaria parasite detection increases during pregnancy in wild chimpanzees. Malar J.

[4] Jeffares DC, Pain A, Berry A, Cox AV, Stalker J, Ingle CE (2007). Genome variation and evolution of the malaria parasite *Plasmodium falciparum*. Nat Genet.

[5] Rich SM, Leendertz FH, Xu G, LeBreton M, Djoko CF, Aminake MN (2009). The origin of malignant malaria. Proc Natl Acad Sci USA.

[6] Prugnolle F, Durand P, Neel C, Ollomo B, Ayala FJ, Arnathau C, et al. African great apes are natural hosts of multiple related malaria species, including *Plasmodium falciparum*. Proc Natl Acad Sci USA. 2011;107:1458–63. 10.1073/pnas.0914440107 PMC2824423 20133889

[7] Krief S, Escalante AA, Pacheco MA, Mugisha L, André C, Halbwax M (2010). On the diversity of malaria parasites in African apes and the origin of *Plasmodium falciparum* from bonobos. PLoS Pathog.

[8] Martin MJ, Rayner JC, Gagneux P, Barnwell JW, Varki A (2005). Evolution of human-chimpanzee differences in malaria susceptibility: relationship to human genetic loss of N-glycolylneuraminic acid. Proc Natl Acad Sci USA.

[9] Pacheco MA, Cranfield M, Cameron K, Escalante AA (2013). Malaria parasite diversity in chimpanzees: the value of comparative approaches to ascertain the evolution of *Plasmodium falciparum* antigens. Malar J.

[10] Liu W, Sundararaman SA, Loy DE, Learn GH, Li Y, Plenderleith LJ (2016). Multigenomic delineation of *Plasmodium* species of the *Laverania* subgenus infecting wild-living chimpanzees and gorillas. Genome Biol Evol.

[11] Liu W, Li Y, Learn GH, Rudicell RS, Robertson JD, Keele BF (2010). Origin of the human malaria parasite *Plasmodium falciparum* in gorillas. Nature.

[12] Hughes AL, Verra F (2010). Malaria parasite sequences from chimpanzee support the co-speciation hypothesis for the origin of virulent human malaria (*Plasmodium falciparum*). Mol Phylogenet Evol.

[13] Silva JC, Egan A, Arze C, Spouge JL, Harris DG (2015). A new method for estimating species age supports the co-existence of malaria parasites and their mammalian hosts. Mol Biol Evol.

[14] Prugnolle F, Durand P, Ollomo B, Duval L, Ariey F, Arnathau C (2011). A fresh look at the origin of *Plasmodium falciparum*, the most malignant malaria agent. PLoS Pathog.

[15] Zerka A, Kaczmarek R, Jaskiewicz E (2015). From malaria parasite point of view-*Plasmodium falciparum* evolution. Postepy Hig Med Dośw Online.

[16] Otto TD, Rayner JC, Böhme U, Pain A, Spottiswoode N, Sanders M (2014). Genome sequencing of chimpanzee malaria parasites reveals possible pathways of adaptation to human hosts. Nat Commun.

[17] Yasukochi Y, Naka I, Patarapotikul J, Hananantachai H, Ohashi J (2017). Evolution of Fseg/Cseg dimorphism in region III of the *Plasmodium falciparum* eba-175 gene. Infect Genet Evol.

[18] Tham W-H, Healer J, Cowman AF (2012). Erythrocyte and reticulocyte binding-like proteins of *Plasmodium falciparum*. Trends Parasitol.

[19] Beeson JG, Drew DR, Boyle MJ, Feng G, Fowkes FJI, Richards JS (2016). Merozoite surface proteins in red blood cell invasion, immunity and vaccines against malaria. FEMS Microbiol Rev.

[20] Adams JH, Blair PL, Kaneko O, Peterson DS (2001). An expanding ebl family of *Plasmodium falciparum*. Trends Parasitol.

[21] Network MGE (2015). A novel locus of resistance to severe malaria in a region of ancient balancing selection. Nature.

[22] Lisowska E (1988). Antigenic properties of human erythrocyte glycophorins. Adv Exp Med Biol.

[23] Tolia NH, Enemark EJ, Sim BKL, Joshua-Tor L (2005). Structural basis for the EBA-175 erythrocyte invasion pathway of the malaria parasite *Plasmodium falciparum*. Cell.

[24] Wanaguru M, Crosnier C, Johnson S, Rayner JC, Wright GJ (2013). Biochemical analysis of the *Plasmodium falciparum* erythrocyte-binding antigen-175 (EBA175)-glycophorin a interaction: implications for vaccine design. J Biol Chem.

[25] Salinas ND, Paing MM, Tolia NH (2014). Critical glycosylated residues in exon three of erythrocyte glycophorin a engage *Plasmodium falciparum* EBA-175 and define receptor specificity. MBio.

[26] Thompson JK, Triglia T, Reed MB, Cowman AFA (2001). Novel ligand from *Plasmodium falciparum* that binds to a sialic acid-containing receptor on the surface of human erythrocytes. Mol Microbiol.

[27] Narum DL, Fuhrmann SR, Luu T, Sim BKL (2002). A novel *Plasmodium falciparum* erythrocyte binding protein-2 (EBP2/BAEBL) involved in erythrocyte receptor binding. Mol Biochem Parasitol.

[28] Gilberger T-W, Thompson JK, Triglia T, Good RT, Duraisingh MT, Cowman AFA (2003). Novel erythrocyte binding antigen-175 paralogue from *Plasmodium falciparum* defines a new trypsin-resistant receptor on human erythrocytes. J Biol Chem.

[29] Lobo C-A, Rodriguez M, Reid M, Lustigman S, Glycophorin C (2003). Is the receptor for the *Plasmodium falciparum* erythrocyte binding ligand PfEBP-2 (baebl). Blood.

[30] Maier AG, Duraisingh MT, Reeder JC, Patel SS, Kazura JW, Zimmerman PA (2003). *Plasmodium falciparum* erythrocyte invasion through glycophorin C and selection for Gerbich negativity in human populations. Nat Med.

[31] Jiang L, Duriseti S, Sun P, Miller LH (2009). Molecular basis of binding of the *Plasmodium falciparum* receptor BAEBL to erythrocyte receptor glycophorin C. Mol Biochem Parasitol.

[32] Rydzak J, Kaczmarek R, Czerwinski M, Lukasiewicz J, Tyborowska J, Szewczyk B (2015). The baculovirus-expressed binding region of *Plasmodium falciparum* EBA-140 ligand and its glycophorin C binding specificity. PLoS One.

[33] Mayer DCG, J-B M, Feng X, Su X, Miller LH (2002). Polymorphism in a *Plasmodium falciparum* erythrocyte-binding ligand changes its receptor specificity. J Exp Med.

[34] Rydzak J, Kmiecik AM, Jaśkiewicz E (2013). Human erythrocyte glycophorin C as the receptor for EBA-140 *Plasmodium falciparum* merozoite ligand. Postepy Hig Med Dosw.

[35] Malpede BM, Lin DH, Tolia NH (2013). Molecular basis for sialic acid-dependent receptor recognition by the *Plasmodium falciparum* invasion protein erythrocyte-binding antigen-140/BAEBL. J Biol Chem.

[36] Mayer DCG, Jiang L, Achur RN, Kakizaki I, Gowda DC, Miller LH. The glycophorin C N-linked glycan is a critical component of the ligand for the *Plasmodium falciparum* erythrocyte receptor BAEBL. Proc Natl Acad Sci USA. 2006;103:2358–62. 10.1073/pnas.0510648103 PMC1413722 16461900

[37] Rayner JC, Huber CS, Galinski MR, Barnwell JW (2004). Rapid evolution of an erythrocyte invasion gene family: the *Plasmodium reichenowi* reticulocyte binding like (RBL) genes. Mol Biochem Parasitol.

[38] Rayner JC, Huber CS, Barnwell JW (2004). Conservation and divergence in erythrocyte invasion ligands: *Plasmodium reichenowi* EBL genes. Mol Biochem Parasitol.

[39] Varki A, Gagneux P (2009). Human-specific evolution of sialic acid targets: explaining the malignant malaria mystery?. Proc Natl Acad Sci USA.

[40] Wanaguru M, Liu W, Hahn BH, Rayner JC, Wright GJ. RH5-basigin interaction plays a major role in the host tropism of *Plasmodium falciparum*. Proc Natl Acad Sci USA. 2013;110:20735–40. 10.1073/pnas.1320771110 PMC3870751 24297912

[41] Wilder JA, Hewett EK, Gansner ME (2009). Molecular evolution of GYPC: evidence for recent structural innovation and positive selection in humans. Mol Biol Evol.

[42] Le Van Kim C, Piller V, Cartron JP, Colin Y, Glycophorins C (1996). D are generated by the use of alternative translation initiation sites. Blood.

[43] Ashline DJ, Duk M, Lukasiewicz J, Reinhold VN, Lisowska E, Jaskiewicz E (2015). The structures of glycophorin C N-glycans, a putative component of the GPC receptor site for *Plasmodium falciparum* EBA-140 ligand. Glycobiology.

[44] Zerka A, Olechwier A, Rydzak J, Kaczmarek R, Jaskiewicz E (2016). Baculovirus-expressed *Plasmodium reichenowi* EBA-140 merozoite ligand is host specific. Parasitol Int.

[45] Zerka A, Rydzak J, Lass A, Szostakowska B, Nahorski W, Wroczyńska A (2015). Studies on immunogenicity and antigenicity of baculovirus-expressed binding region of *Plasmodium falciparum*. Arch Immunol Ther Exp.

[46] Jaskiewicz E, Czerwinski M, Colin Y, Lisowska E (2002). Recombinant forms of Gerbich blood group antigens: expression and purification. Transfus Clin Biol J Societe Fr Transfus Sang.

[47] Laemmli UK (1970). Cleavage of structural proteins during the assembly of the head of bacteriophage T4. Nature.

[48] Towbin H, Staehelin T, Gordon J (1979). Electrophoretic transfer of proteins from polyacrylamide gels to nitrocellulose sheets: procedure and some applications. Proc Natl Acad Sci USA.

[49] Jaskiewicz E, Czerwinski M, Uchikawa M, Murata S, Miyazaki T, Ikeda H (2002). Recombinant forms of glycophorin C as a tool for characterization of epitopes for new murine monoclonal antibodies with anti-glycophorin C specificity. Transfus Med.

[50] Duah NO, Matrevi SA, Quashie NB, Abuaku B, Koram KA (2016). Genetic diversity of *Plasmodium falciparum* isolates from uncomplicated malaria cases in Ghana over a decade. Parasit Vectors.

[51] Corbel V, Henry M-C (2011). Prevention and control of malaria and sleeping sickness in Africa: where are we and where are we going?. Parasit Vectors.

